# Effects of a Pragmatic Lifestyle Intervention for Reducing Body Mass in Obese Adults with Obstructive Sleep Apnoea: A Randomised Controlled Trial

**DOI:** 10.1155/2014/102164

**Published:** 2014-07-21

**Authors:** James Moss, Garry Alan Tew, Robert James Copeland, Martin Stout, Catherine Grant Billings, John Michael Saxton, Edward Mitchell Winter, Stephen Mark Bianchi

**Affiliations:** ^1^The Farr Institute of Health Informatics, Department of Epidemiology and Public Health, University College London, 222 Euston Road, London NW1 2DA, UK; ^2^York Trials Unit, University of York, Heslington, York YO10 5DD, UK; ^3^Centre for Sport and Exercise Science, Sheffield Hallam University, Sheffield S10 2BP, UK; ^4^North West Heart Centre, Wythenshawe Hospital, University Hospital of South Manchester, Southmoor Road, Manchester M23 9LT, UK; ^5^Academic Directorate of Respiratory Medicine, Sheffield Teaching Hospitals Foundation Trust, Sheffield S5 7AU, UK; ^6^School of Health Sciences, University of East Anglia, Norwich Research Park, Norwich NR4 7TJ, UK

## Abstract

This study investigated the effects of a pragmatic lifestyle intervention in obese adults with continuous positive airway pressure-treated obstructive sleep apnoea hypopnoea syndrome (OSAHS). Sixty patients were randomised 1 : 1 to either a 12-week lifestyle intervention or an advice-only control group. The intervention involved supervised exercise sessions, dietary advice, and the promotion of lifestyle behaviour change using cognitive-behavioural techniques. Outcomes were assessed at baseline (week 0), intervention end-point (week 13), and follow-up (week 26). The primary outcome was 13-week change in body mass. Secondary outcomes included anthropometry, blood-borne biomarkers, exercise capacity, and health-related quality of life. At end-point, the intervention group exhibited small reductions in body mass (−1.8 [−3.0, −0.5] kg; *P* = 0.007) and body fat percentage (−1 [−2, 0]%; *P* = 0.044) and moderate improvements in C-reactive protein (−1.3 [−2.4, −0.2] mg*·*L^−1^; *P* = 0.028) and exercise capacity (95 [50, 139] m; *P* < 0.001). At follow-up, changes in body mass (−2.0 [−3.5, −0.5] kg; *P* = 0.010), body fat percentage (−1 [−2, 0]%; *P* = 0.033), and C-reactive protein (−1.3 [−2.5, −0.1] mg*·*L^−1^; *P* = 0.037) were maintained and exercise capacity was further improved (132 [90, 175] m; *P* < 0.001). This trial is registered with ClinicalTrials.gov NCT01546792.

## 1. Introduction

Obstructive sleep apnoea hypopnoea syndrome (OSAHS) is the most common form of sleep-disordered breathing, characterised by repetitive nocturnal airway obstruction and frequent nocturnal arousal from sleep that leads to excessive daytime sleepiness (EDS). Prevalence surveys suggest that 2% of women and 4% of men at middle age are affected by this syndrome, which is becoming increasingly common with the current obesity epidemic [1]. The clinical consequences of repeated airway closures (hypoxaemia, sympathoexcitation, and oxidative stress) contribute to the premature development of cardiovascular disease, specifically ischaemic heart disease, stroke, and hypertension. Individuals with OSAHS are often obese [2] and physically inactive [3], and epidemiological data indicate an independent relationship between OSAHS and cardiovascular disease [4]. Obesity is a key modifiable risk factor for OSAHS [5] with recent guidelines suggesting that all overweight and obese patients with OSAHS be encouraged to lose weight [6]. As both untreated OSAHS and obesity contribute to the increased morbidity and mortality, interventions capable of addressing both should be considered.

Continuous positive airway pressure (CPAP), the primary therapy for moderate-to-severe OSAHS, improves subjective and objective measures of sleepiness [7] but provides only day-to-day management of the condition, not a long-term cure (i.e., withdrawal of CPAP causes symptoms to return). Moreover, CPAP has minimal effects on patients' weight or physical activity levels [8] despite increasing exercise capacity [9]. In contrast, intensive lifestyle interventions, which typically involve very low energy diets (VLEDs), appear to be effective for initial rapid weight reduction and can sometimes result in complete remission of OSAHS [10, 11], although steady postintervention weight regaining is common [12–14]. However, intensive lifestyle interventions are limited in that they might not be acceptable to many patients or deliverable within a tax-funded healthcare system. Further research is needed to explore the impact of lower-intensity (i.e., more practical) lifestyle interventions on body mass (i.e., weight) and other important health outcomes in overweight and obese individuals with OSAHS.

We conducted a randomised controlled trial to determine the impact of a 12-week pragmatic lifestyle intervention on body mass and other indicators of health and fitness in obese adults who were currently being treated with CPAP for moderate-to-severe OSAHS. The pragmatic lifestyle intervention incorporated supervised exercise sessions, dietary education and advice, and the promotion of lifestyle behaviour change using cognitive-behavioural techniques. We hypothesised that the intervention group would have greater improvement in body mass and other cardiometabolic outcomes, compared with an advice-only control group.

## 2. Materials and Methods

### 2.1. Participants

Patients with OSAHS were recruited from sleep clinics at Sheffield Teaching Hospitals NHS Foundation Trust. Eligible patients were obese (body mass index (BMI) > 30 kg*·*m^−2^) men and women aged 18–85 years with at least moderate OSAHS (apnoea hypopnoea index (AHI) > 15 events*·*h^−1^; oxygen desaturation index > 15 events*·*h^−1^; Epworth Sleepiness Scale (ESS) >11) treated with CPAP therapy. Adherence was assessed subjectively using self-report during history taking (>75% nightly use; >4 hours per night) and objectively by percentage of nights CPAP was used combined with indices of treatment efficacy (i.e., normalisation of AHI and ESS). Exclusion criteria were any contraindications to exercise testing and training such as severe hypertension, unstable angina, uncontrolled cardiac arrhythmias, and inability or unwillingness to undertake the commitments of the study or participation in regular purposeful exercise (>30 min, ≥3 times per week; self-reported). The study was approved by the South Yorkshire Research Ethics Committee (09/H1310/74) and all participants provided written informed consent prior to enrolment.

### 2.2. Sample Size

The primary outcome measure was body mass (at intervention end-point) because weight loss is a key focus of management guidelines for overweight and obese individuals with OSAHS [6], and previous studies have shown reductions in body mass correlate with improvements in severity of OSAHS [10, 15]. A total of 60 participants (30 per group) were required to detect a between-group difference of at least 1.5 kg at intervention end-point, assuming a standard deviation of 12 kg for body mass [16], a pre-post correlation for body mass of 0.991 [17], 20% attrition, 90% power, and a 2-tailed alpha of 0.05.

### 2.3. Design and Randomisation

This was a nonblinded, parallel-group, randomised controlled trial. Participants were allocated 1 : 1 to either a 12-week pragmatic lifestyle intervention or an advice-only control group using a randomisation sequence created by an independent researcher prior to recruitment (nQuery, Statistical Solutions, USA). The research team were notified of group allocation once each participant completed their baseline assessment. Outcome measures were assessed before randomisation (week 0), at intervention end-point (week 13), and after 13 weeks of independence (week 26).

### 2.4. Pragmatic Lifestyle Intervention

Participants randomised to the pragmatic lifestyle intervention were invited to attend supervised exercise sessions at a university exercise facility within one mile of the treating hospital. The frequency of exercise sessions was initially three per week. This was reduced to two per week during weeks 5 to 8 (with a third exercise session undertaken independently by participants at their convenience) and then to one per week (with two self-directed exercise sessions) during weeks 9 to 12. This pattern was designed to gradually decrease dependence on our facility, supervision, and expertise and to promote participants' independent exercise participation, which was reported in an exercise diary. Exercise sessions lasted approximately one hour and typically comprised 45 minutes of aerobic interval training (treadmill walking/jogging, cycling, and rowing), 15 minutes of resistance training (major muscle groups), and exercises aimed at improving flexibility and balance. The aerobic interval training involved alternating hard and easy exercise bouts at a ratio of 1 : 2 (e.g., 0.5 min hard, 1 min easy), progressing to 4 : 1 (e.g., 4 min hard, 1 min easy) as tolerated. Heart rate and perceived exertion (RPE, using a 6 to 20 scale; Borg, 1982) were recorded at the end of hard intervals to facilitate prescription and monitor progression (RPE: 14–16 for hard bouts and 9–11 for easy bouts). Exercise sessions were individualised and directed by an exercise physiologist taking into account participants' health, mobility, and preferences. The cognitive-behavioural component of the intervention was integrated into exercise sessions and involved psychoeducation (based upon attitudes, experiences, emotions, beliefs, etc.) tailored to participants' stage of change and implemented the cognitive-behavioural processes of change as outlined by the transtheoretical model [18]. Such an approach has demonstrated efficacy in clinical populations previously [19]. Concurrent dietary education and advice based on the principles of the eatwell plate model (http://www.eatwell.gov.uk) were also integrated into the sessions. A three-day diet diary was completed and assessed to identify dietary imbalance and as a tool to set short- and long-term goals. A British Heart Foundation (BHF) weight loss leaflet “So… you want to lose weight for good?” was provided and key concepts were extracted from it. Participants allocated to the advice-only control group received a letter explaining their group allocation, basic written lifestyle advice, and the BHF weight loss leaflet.

### 2.5. Study Outcomes

Participants' body mass was measured in duplicate using a calibrated beam-balance scale (Model 424; Weylux; Hallamshire Scales Ltd., Sheffield, UK). Participants were minimally dressed and the mean of two consecutive concordant measurements was used. Secondary outcomes were BMI; neck, waist, and hip circumferences; body fat percentage (Bodystat Quadscan 4000, Bodystat Ltd., IM99 1DQ); health-related quality of life using the EuroQol EQ5D-3L questionnaire (EuroQol Executive Office, 3068 AV Rotterdam, Netherlands); and exercise capacity using the incremental shuttle walking test (ISWT; [20]). Fasting venous blood was collected, centrifuged, separated, and frozen for subsequent biochemical analysis. Full lipid profile, glucose, and high-sensitivity C-reactive protein (hs-CRP) were measured on ADVIA 2400 and insulin concentrations on ADVIA Centaur XP (Siemens, 511 Benedict Avenue, Tarrytown, NY).

### 2.6. Statistical Analyses

The mean difference in change of body mass between the treatment groups was assessed at intervention end-point (week 13) by analysis of covariance (ANCOVA) using baseline body mass as a covariate and change scores (end-point minus baseline) as the dependent variable. The adjusted mean difference in change between groups at week 13 and corresponding 95% confidence interval (CI) from the model are presented. All analyses were done on an intention-to-treat basis with previous observations carried forward where necessary. The same procedure was used to assess treatment difference in body mass at follow-up (week 26). Treatment differences for other outcomes were similarly analysed using separate ANCOVAs for intervention end-point (week 13) and follow-up (week 26). All analyses were carried out in SPSS version 18.0 (SPSS UK Ltd., 2 New Square (B3 Floor 2), Bedfont Lakes, UK). Statistical tests were at a two-sided 0*·*05 significance level. Analysis of residuals was undertaken for all regression models in order to assess model assumptions.

## 3. Results

### 3.1. Participant Characteristics

Sixty patients with controlled OSAHS (ESS: 5.0 [3.0, 6.8]; AHI: 2.4 [1.9, 3.2] events*·*h^−1^) that were long-term CPAP users enrolled on the study. Due to some patients relocation from hospital trusts elsewhere in the UK, CPAP start dates were only available for 75% of participants. For these patients, median [range] CPAP history was 1.2 [0.5–10.8] years. All sixty patients had a verifiable usage history of at least 6 months. Both groups were classified as obese, normocholesterolaemic, normoglycaemic, and hyperinsulinaemic. Baseline characteristics are summarised in Table 1.

### 3.2. Recruitment, Retention, and Compliance

**Figure 1:**
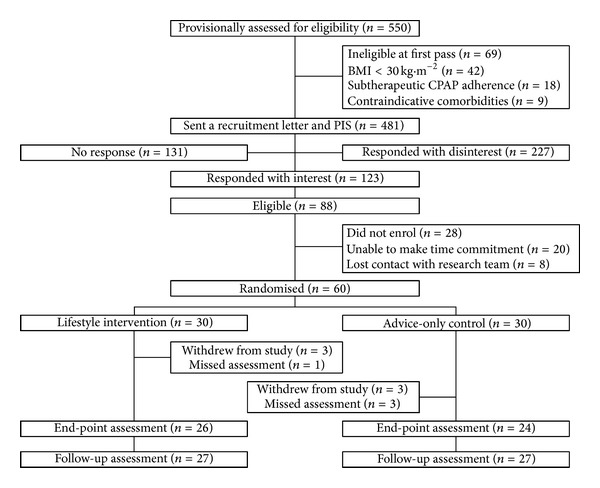
CONSORT flow-chart.

We invited 481 potentially eligible patients to participate in our study, of which 123 (26%) responded with interest and underwent further screening (Figure 1). Of these, 60 (49%) enrolled giving a recruitment rate of 12%. Although six participants (10%) withdrew from the study (unrelated health change: *n* = 2; change in work commitments: *n* = 2; no reason: *n* = 2), 97% of assessments (157 of 162) and 96% of exercise sessions (620 of 648) were attended. No adverse events were recorded in more than 650 hours of exercise training.

### 3.3. Anthropometrics

End-point and follow-up data for anthropometric outcomes are presented in Table 2. The adjusted mean difference in change in body mass at intervention end-point (primary outcome) was −1.8 [−3.0, −0.5] kg (*P* = 0.007), favouring the intervention group. A similar difference was maintained at follow-up (−2.0 [−3.5, −0.5] kg; *P* = 0.010). These differences were accompanied by −0.8 [−1.3, −0.3] and −0.9 [−1.5, −0.3] kg*·*m^−2^ changes in BMI (*P* = 0.002 and *P* = 0.002, resp.) and −1 [−2, 0] and −1 [−2, 0] % changes in body fat percentage (*P* = 0.044 and *P* = 0.033, resp.). There were no significant differences in other anthropometric variables.

### 3.4. Exercise Capacity

Although data were collected for all participants, seven ISWT datasets (including ISWD and postexercise heart rate (HR), systolic blood pressure (SBP), and diastolic blood pressure (DBP)) were excluded because participants completed the 1020-metre test at one or more time-points (four at baseline and three at end-point). This ceiling effect was unexpected and dilutes the true treatment effect; consequently these patients were excluded from the analysis.

Despite secure randomisation there was a large chance imbalance between groups at baseline for distance walked in the ISWT (475 ± 240 versus 639 ± 198 m). Nevertheless, the adjusted difference in change favoured the intervention group at end-point (95 [50, 139] m; *P* < 0.001) and follow-up (132 [90, 175] m; *P* < 0.001) at follow-up. This observation occurred in the absence of any significant changes in any postexercise physiological (SBP, DBP, and HR) or psychophysiological (RPE) measures (all *P* > 0.05).

### 3.5. Biomarkers

Nine patients were unable to provide a venous blood sample at the baseline assessment. To prevent potential confounding from acute inflammation, eight datasets were excluded because CRP measurement exceeded 10 mg*·*L^−1^ [21]. Forty-three complete datasets remained (control: *n* = 21; intervention: *n* = 22). There was no change in serum cholesterol, triglycerides, HDL, LDL, or glucose at end-point or follow-up. Change in hs-CRP at end-point favoured the intervention group (−1.3 [−2.4, − 0.2] mg*·*L^−1^, *P* = 0.028), and this improvement was maintained at follow-up (−1.3 [−2.5, − 0.1] mg*·*L^−1^, *P* = 0.037). There was no evidence of any significant changes in serum insulin over any of the time periods.

### 3.6. Quality of Life

There was a reduced proportion of participants in the intervention group reporting problems in performing usual activities (e.g., vacuuming, cleaning, and shopping) at end-point (*P* = 0.044) but not follow-up (*P* = 0.375). There were no changes in the other four EQ5D domains. Although there was no significant change in self-perceived health score (i.e., EQ Visual Analogue Scale) at end-point, there was significant improvement at follow-up of 9 [2, 16] points (*P* = 0.017).

## 4. Discussion

This study demonstrates that a pragmatic lifestyle intervention focused on introducing structured exercise, providing dietary advice, and combining both with behaviour change counselling improves body mass, exercise capacity, and a marker of systemic inflammation in obese adults who were being treated with CPAP for OSAHS. The programme was well tolerated by patients, exemplified by excellent attendance rates and no reported adverse events (in more than 650 hours of exercise testing and training).

Although the intervention favoured reductions in body mass aligned with our hypothesis, the magnitude of these changes (approx. 2 kg loss at both time-points) was small and possibly inadequate to provide any clinical benefit. Body mass reductions in interventions incorporating hypoenergetic diets are typically greater [22, 23] than those without [24, 25]. VLEDs are often supplemented with psychological and dietary support, which rarely alleviates the steady postintervention weight regain often observed with these interventions [12, 14]. It is likely that the severe nature of such interventions produces a rapid weight loss that is unsustainable in the longer term. Our intervention was designed and used on the premise that a pragmatic approach based on a slower rate of weight loss induced by making smaller changes to behaviour and practising a “healthy lifestyle” could offer more sustainable long-term benefit.

Recent meta-analyses have collated evidence on dietary interventions [26] and exercise-based interventions [27] in OSAHS. The former reported a weighted mean reduction in BMI of 4.8 [3.8, 5.9] kg*·*m^−2^ and in AHI of 23.1 [8.9, 37.3] events*·*h^−1^. The results of exercise-based interventions appeared less effective, with mean reductions of 1.4 [−2.8, − 0.1] kg*·*m^−2^ and 6.3 [−8.5, − 4.0] events*·*h^−1^ for BMI and AHI, respectively [27]. However, these latter changes in BMI and AHI included a study [13] that incorporated a proprietary VLED alongside the exercise component; this study alone had a BMI reduction almost double that of the five other included studies combined (−6.0 versus −3.3 (*n* = 5) kg*·*m^−2^) which distorted the average effect. Despite the greater weight loss, the improvement in AHI was smaller than 4 of the 5 other exercise trials. Furthermore, the Barnes et al. study was a noncontrolled nonrandomised design. The changes in BMI reported in the current study better match those in exercise-only trials. The 15 [8, 22] and 21 [14, 27]% improvements in ISWD at end-point and follow-up are consistent with improvements reported by others [13, 28] and in our study come atop of any improvement that can be observed through CPAP therapy alone [9]. Blair [29] suggested that low cardiorespiratory fitness was attributable to a greater proportion of all-cause mortality (4000 deaths) than obesity, smoking, and hypercholesterolaemia combined. This evidence amplifies the importance of improving fitness, even in the absence of changes in “fatness,” in clinical populations.

Evidence suggests that addressing energy intake is more efficacious for weight loss than increasing energy expenditure [30] and that exercise trials that provide dietary advice only are associated with smaller weight losses than those providing a VLED [26, 27]. Although we acknowledged this when designing the study, we feel the severe rate of weight loss, and typical weight regain seen over time is not pragmatic enough for introduction into current healthcare delivery. The premise of making smaller changes in dietary behaviours that are more sustainable in the long term still merits investigation and is now supported by the National Institute for Health and Care Excellence [31] in the UK as clinically beneficial and cost-effective. All components of the current intervention were delivered by the same investigator (JM), who used psychological techniques (such as motivational interviewing) to elicit core behaviours and previous barriers to behaviour change in order to customise the intervention delivered to promote longer-term adherence.

Hs-CRP is a systemic marker of inflammation strongly associated with atherosclerotic plaque development, cardiovascular disease risk, and death. Our intervention demonstrated a significant improvement in hs-CRP at end-point (−1.3 mg*·*L^−1^) that was maintained at follow-up. It has been shown previously that circulating CRP is elevated in OSAHS and that effective CPAP therapy can normalise this augmentation [32]. Furthermore, there is evidence to suggest that exercise can have a beneficial effect on hs-CRP concentration in humans [33]. As our patients were already compliant with CPAP therapy, any further reduction in CRP could reflect further decrement in cardiovascular disease risk and progression.

Improvements in exercise capacity were not surprising findings considering the design of our study. The key finding here was that, after 13 weeks of independence, improvements in exercise capacity were not only maintained but further improved upon. Although we included no measure of exercise behaviour during the independence period, further improvements are probably reflective of continued regular exercise training, which was mentioned anecdotally by participants during assessments.

*Limitations*. Although a metric of OSAHS severity was not included in the current study for financial and logistical reasons, it was thought that changes in body mass could be a surrogate marker for changes in OSAHS severity. Improvements in OSAHS in the absence of weight loss have been demonstrated by other groups, coupled with evidence that sedentary behaviour and low cardiorespiratory fitness could pose a greater public health concern than obesity, suggests that the improvements in exercise capacity demonstrated in the current study should not be underestimated. Although the current study is powered for changes in body mass, we do not know if it was adequately powered for the secondary outcome measures, so these findings must be interpreted with caution. Other limitations include the single-centre study design and nonblinded assessments, which were unavoidable because of resource limitation. However, we have demonstrated an acceptable and deliverable programme with positive outcomes that should, in combination with enhanced dietary intervention, be further investigated in a larger OSAHS group.

## 5. Conclusion

A pragmatic lifestyle intervention in OSAHS has been shown to improve cardiometabolic outcomes in obese adults treated with CPAP for OSAHS. This approach is one that could be deliverable within the UK (and other) healthcare systems. Further research is required to further investigate the clinical efficacy and cost-effectiveness of pragmatic lifestyle interventions in OSAHS.

## Figures and Tables

**Table 1:** Baseline group characteristics.

Variable	Control		Intervention	
	*n*		*n*	
Anthropometry				
Body mass (kg)	30	118.3 ± 21.9	30	117.4 ± 24.3
Body mass index (kg*·*m^−2^)	30	39.8 ± 7.0	30	38.9 ± 6.9
Body fat (%)	30	40 ± 9	30	39 ± 8
Neck circumference (cm)	30	45 ± 5	30	44 ± 4
Waist circumference (cm)	30	128 ± 14	30	125 ± 16
Hip circumference (cm)	30	129 ± 14	30	125 ± 15
Cardiometabolic				
Resting heart rate (bpm)	30	65 ± 14	30	67 ± 11
Resting systolic BP (mmHg)	30	127 ± 11	30	132 ± 15
Resting diastolic BP (mmHg)	30	72 ± 8	30	75 ± 9
Serum cholesterol (mmol*·*L^−1^)	21	4.8 [3.4, 6.2]	22	4.7 [3, 6.5]
Serum HDL (mmol*·*L^−1^)	21	1.2 ± 0.2	22	1.3 ± 0.3
Cholesterol to HDL ratio	21	4.0 ± 0.7	22	4.1 ± 0.7
Serum triglycerides (mmol*·*L^−1^)	21	1.7 [0.6, 2.8]	22	1.8 [1.2, 2.4]
Serum LDL (mmol*·*L^−1^)	21	2.7 ± 0.7	22	2.9 ± 1.1
Serum CRP (mg*·*L^−1^)	21	2.9 [0.3, 5.5]	22	2 [−0.1, 4.0]
Serum glucose (mmol*·*L^−1^)	21	5.7 ± 1.6	22	5.0 ± 1.1
Serum insulin (mU*·*L^−1^)	20	29 [7, 51]	20	27 [4, 50]
ISWT				
ISWD (m)	27	475 ± 240	26	639 ± 198
Post-ex RPE	27	15 ± 2	26	15 ± 2
Post-ex heart rate (bpm)	27	131 ± 28	26	148 ± 21
Post-ex systolic BP (mmHg)	27	169 ± 29	25	192 ± 31
Post-ex diastolic BP (mmHg)	27	86 ± 15	25	93 ± 11
Quality of life				
EuroQol EQ5D-3L VAS	30	58 ± 18	30	64 ± 17

Data are presented as mean ± SD or median [IQR]. BP: blood pressure; HDL: high-density lipoprotein; LDL: low-density lipoprotein; CRP: C-reactive protein; ISWT: incremental shuttle walk test; ISWD: incremental shuttle walk distance; RPE: Borg rating of perceived exertion; VAS: Visual Analogue Scale.

**Table 2:** Raw data by groups and adjusted mean differences in change at intervention end-point and followup.

Variable	Intervention end-point				Followup			
	(week 13)				(week 26)			
	Control	Intervention	Adjusted mean diff. (95% CI)	P*	Control	Intervention	Adjusted mean diff. (95% CI)	P*
Anthropometry								
Body mass (kg)	117.9 ± 21.0	115.2 ± 24.3	−1.8 (−3.0, −0.5)	0.006	118.1 ± 21.0	115.1 ± 24.4	−2.0 (−3.5, −0.5)	0.010
Body mass index (kg*·*m^−2^)	39.8 ± 6.8	38.0 ± 6.9	−0.8 (−1.3, −0.3)	0.002	39.8 ± 6.7	37.9 ± 6.9	−0.9 (−1.5, −0.3)	0.002
Body fat (%)	40 ± 9	37 ± 8	−1 (−2, 0)	0.044	40 ± 9	38 ± 8	−1 (−2, 0)	0.033
Hip circumference (cm)	128 ± 15	123 ± 15	−1 (−2, 0)	0.093	128 ± 15	122 ± 15	−2 (−3, 0)	0.020
Waist circumference (cm)	127 ± 15	123 ± 16	−2 (−4, 0)	0.117	127 ± 15	123 ± 15	−2 (−4, 1)	0.143
Hip circumference (cm)	128 ± 15	123 ± 15	−1 (−2, 0)	0.093	128 ± 15	122 ± 15	−2 (−3, 0)	0.020
Cardiometabolic								
Resting heart rate (beats*·*min^−1^)	66 ± 12	64 ± 12	−5 (−9, −2)	0.002	62 ± 9	63 ± 10	−2 (−6, 1)	0.165
Resting systolic BP (mmHg)	127 ± 13	130 ± 13	0 (−5, 4)	0.893	131 ± 14	133 ± 16	−2 (−7, 4)	0.534
Resting diastolic BP (mmHg)	72 ± 8	74 ± 9	−1 (−4, 2)	0.590	75 ± 8	76 ± 10	−1 (−4, 2)	0.539
Serum cholesterol (mmol*·*L^−1^)	4.8 [3.2, 6.4]	4.8 [3.5, 6.1]	0.1 (−0.2, 0.3)	0.575	4.4 [2.5, 6.3]	4.6 [3.6, 5.5]	−0.1 (−0.5, 0.3)	0.645
Serum HDL (mmol*·*L^−1^)	1.2 ± 0.2	1.2 ± 0.3	0.0 (−0.1, 0.9)	0.569	1.2 ± 0.3	1.2 ± 0.2	−0.1 (−0.2, 0)	0.241
Serum LDL (mmol*·*L^−1^)	2.7 ± 0.8	2.9 ± 1	0 (−0.2, 0.3)	0.731	2.6 ± 0.9	2.8 ± 0.9	0 (−0.3, 0.3)	0.814
Serum CRP (mg*·*L^−1^)	3.3 [0.1, 6.5]	1.4 [−0.7, 3.5]	−1.3 (−2.4, −0.2)	0.028	3.2 [−2.1, 8.5]	1.8 [0.6, 3]	−1.3 (−2.5, −0.1)	0.037
Serum glucose (mmol*·*L^−1^)	5.7 ± 1.9	4.8 ± 0.5	−0.3 (−0.9, 0.2)	0.224	5.7 ± 1.9	4.8 ± 0.8	−0.3 (−0.9, 0.3)	0.267
ISWT								
ISWD (m)	475 ± 250	724 ± 193	95 (50, 139)	<0.001	471 ± 240	737 ± 179	132 (90, 175)	<0.001
Quality of life								
EQVAS	63 ± 19	60 ± 20	3 (−4, 10)	0.385	69 ± 18	72 ± 16	9 (2, 16)	0.017

Data are presented as mean ± SD or median [IQR]. BP: blood pressure; HDL: high-density lipoprotein; LDL: low-density lipoprotein; CRP: C-reactive protein; ISWT: incremental shuttle walk test; ISWD: incremental shuttle walk distance; RPE: Borg rating of perceived exertion; EQVAS: EuroQol Visual Analogue Scale; P*: *P* value adjusted for baseline score.
